# Monoglyceride Blend Reduces Mortality, Improves Nutrient Digestibility, and Intestinal Health in Broilers Subjected to Clinical Necrotic Enteritis Challenge

**DOI:** 10.3390/ani11051432

**Published:** 2021-05-17

**Authors:** Alip Kumar, Sarbast K. Kheravii, Lily Li, Shu-Biao Wu

**Affiliations:** 1 School of Environmental and Rural Science, University of New England, Armidale, NSW 2351, Australia; akumar26@myune.edu.au (A.K.); sqassim2@une.edu.au (S.K.K.); 2 BASF Animal Nutrition, Asia Pacific, 7 Temasek Boulevard, Singapore 038987, Singapore; lily.d.li@basf.com

**Keywords:** monoglyceride blend, alternatives to antibiotics, performance, intestinal health, clinical necrotic enteritis, broiler chickens

## Abstract

**Simple Summary:**

Necrotic enteritis (NE) is a common and devastating enteric bacterial disease prevalent in fast-growing broilers. It is a great concern to the global poultry industry as impaired performance and high flock mortality up to 50% in severe cases occur, leading to losses of over US$6 billion each year. Controlling NE in fast-growing broilers is crucial, particularly in the antibiotic-free era. Among many potential alternatives to in-feed antibiotics, fatty acid glycerides and formic acid supplementation in diets have shown promising effects in improving performance and intestinal health in broilers infected with subclinical NE. However, data are limited in clinical NE infected broilers. Thus, this study was conducted to evaluate the potential of monoglyceride blend (MG) and buffered formic acid (FA) as alternatives to antibiotics in the performance and intestinal health of broilers subjected to clinical NE challenge. The obtained results highlighted that the diet supplemented with MG has the potential to improve intestinal health and reduce the severity of clinical NE by reducing mortality. This study underpins the importance of additives in poultry production following the removal of antibiotics in poultry feed to alleviate the possible loss posed by enteric diseases such as NE.

**Abstract:**

This study evaluated the potential of monoglyceride blend (MG) and buffered formic acid (FA) as alternatives to antibiotics in the performance and intestinal health of broilers under clinical necrotic enteritis (NE) challenge. A total of 544 as-hatched Ross 308 broiler chicks were randomly distributed to 32-floor pens housing 17 birds per pen. The four treatments were: NC—non-additive control; ZBS—antibiotic group supplemented with zinc bacitracin and salinomycin; MG—additive MG supplementation in the starter phase only; and MGFA—additive MG in starter phase and FA in grower and finisher phases. All birds were challenged with *Eimeria* spp. and *Clostridium perfringens*. Results showed that the NC group had lower BWG and higher FCR than the ZBS group in the grower and overall period (*p* < 0.05). The NC group had higher NE-caused mortality (days 14 to 17) than the ZBS group (*p* < 0.05). Birds fed MG had lower NE-caused mortality than the NC group (*p* < 0.05). Birds fed MG had upregulated jejunal tight junction protein1 (*TJP1*) and immunoglobulin (*IgG*) on day 16 and improved gross energy digestibility on day 24 than the NC group (*p* < 0.05). These findings suggest that supplementation of MG may improve intestinal health and protect birds from clinical NE occurrence.

## 1. Introduction

Necrotic enteritis (NE) is one of the world’s most economically important and severe enteric poultry diseases caused by NetB producing *Clostridium perfringens* [1]. The economic costs to the world poultry industry by NE have been estimated to be over US $6 billion per annum associated with disease control measures and production losses [2]. The NE is typified by reduced body weight gain (BWG), feed intake (FI) and digestibility, increased feed conversion ratio (FCR), intestinal lesions and increased incidence of wet litter, and diarrhea [3,4]. In addition, NE damages intestinal epithelial cells and the mucosa in general, impairing the function of tight junction genes, which leads to a disruption of microbial inhabitants. The acute clinical form of NE can cause sudden death and a high flock mortality rate of 2 to 10% and in severe cases up to 50% over several days, whereas the subclinical form of NE can significantly impair growth performance and reduce FCR [5]. The occurrence and the severity of NE are affected by the presence of predisposing factors (e.g., coccidiosis, fish meal, and poor management) [6,7].

The application of in-feed antibiotic growth promoters (AGP) has been banned in many countries (e.g., European Union or phasing-out worldwide in the poultry industry due to public concerns over bacterial antibiotic resistance). This, in turn, has contributed to a higher prevalence of economically important enteric diseases in livestock such as NE in poultry [8]. Consequently, the poultry industry faces challenges with the health and performance of the birds that have led to increased production costs for disease control and management purposes. Thus, the poultry and other livestock industries are in need of potential in-feed antibiotic alternatives to improve the performance and protect the intestinal health of the birds so as to minimize the production cost and profit losses in the post-antibiotic era.

To achieve improved production in animals, certain bioactive ingredients have been used to supplement diets as alternatives to in-feed AGP. Organic acids (OA) have been used in poultry feed as a preservative over several decades and increased interest in recent years as a possible alternative to in-feed AGP due to its promising effects on bird performance and intestinal health [9,10]. Among the OA, dietary addition of short-chain fatty acids (SCFA) and medium-chain fatty acids (MCFA) in different forms such as salts and glycerides alone and their blends have been used to protect gut barrier integrity and control the balance of microbiota by their bactericidal and bacteriostatic characteristics, resulting in better animal performance [10,11,12]. The efficacy of OA and their blends varies due to the chemical composition, pKa value, form, molecular weight, and experimental conditions [13]. A widely accepted form of OA is glycerides, an esterified product of fatty acids. Fatty acid glycerides are free from unpleasant odors and thus easier to handle. They can be released to the lower part of the digestive tract under their lipase actions. Glyceride products are known to have enhanced antibacterial activities due to the improved availability in the lower part of the intestine [14,15]. Previous studies have shown that the diet supplemented with butyric acid or its glyceride derivatives could replace antibiotics and maintain optimal bird performance [12,16] and reduce *Salmonella enteritidis* caused infection [17]. A recent study has shown that birds fed different dosages of butyric acid glycerides significantly improved overall FCR under subclinical NE challenge [18]. However, other studies have reported inconsistent growth performance results in birds supplemented with butyric acid glycerides alone or in combination with tributyrin, and a combination of mono- and di-glyceride products [19,20]. The types of butyric acid derivatives, forms of delivery, amount of active compounds, dosage, diet composition, management, bird health, and environmental conditions or disease may contribute to the different results observed in the literature. 

Moreover, a recent study reported that the birds fed a monoglyceride blend (MG) at a high dose had improved FCR compared to the challenged control group in the grower phase, but the low dose did not [18]. Buffered formic acid (FA) at a high dose improved FCR in the finisher phase, but had no effect in the starter and grower phases. Birds fed MG at a low dose had improved FCR compared to the birds fed FA at a high dose in the starter phase. Therefore, this study was to investigate whether the supplementation of MG and FA at appropriate doses in different feeding phases may help to achieve optimal bird performance under diseased conditions [18]. It was hypothesized that (a) the supplementation of MG at a high dose in the starter phase improves performance and protects birds from NE in the later phases; and (b) FA supplementation in grower and finisher phases provides additional benefits to improve performance and protect the intestinal health of birds from the negative effects of NE.

The current study was designed to evaluate the effects of MG supplementation in the starter phase and the effects of FA supplementation in grower and finisher phases in the mitigation of NE.

## 2. Materials and Methods

### 2.1. Ethics Statement

The experimental procedures were approved by the Animal Ethics Committee of University of New England, Australia (Approval No.: AEC18-007) and conducted according to the guidelines for the care and use of laboratory and farm animals for scientific purposes accredited by the Australian Bureau of Animal Health [21].

### 2.2. Feed Additives

The current study evaluated the potential of two different types of feed additives supplied by BASF, Germany to improve performance and intestinal health in broilers as antibiotic alternatives using the clinical NE challenge model. The feed additives were: (A) monoglyceride blend (MG), a blend of mono-, di- and tri-glycerides with the main component being 1-monoglycerides (BalanGut^TM^ LS P), primarily composed of approximately 45% mono-, di- and tri-glycerides of butyric, caprylic, and capric acids; and (B) buffered formic acid (FA), primarily composed of approximately 61% formic acid and 20.5% sodium formate (Amasil^®^ NA).

### 2.3. Design and Animal Husbandry

A total of as-hatched 544 mixed-sex Ross 308 broiler chicks were obtained on the day of hatching from Baiada Hatchery in Tamworth, NSW, Australia. Birds were vaccinated against Marek’s disease and infectious bronchitis disease at the hatchery. Upon arrival, the gender of birds was determined by feather sexing and allocated to four treatments in 32-floor pens measuring 75 × 120 cm, based on a completely randomized design (CRD). Each of the four treatment groups had eight replicate pens with 17 birds per pen (eight males and nine females). Birds were raised in an environmentally controlled facility with softwood shavings as litter material. Clean water and feed were provided ad libitum with the temperature, relative humidity, and lighting following Ross 308 guidelines [22].

Birds in all treatment groups were challenged with *Eimeria* spp. and *C. perfringens* as shown in Table 1. 

The treatments were: T1—non-additive control, without additives or in-feed antibiotics (NC); T2—control diet supplemented with in-feed zinc bacitracin (0.033%) and salinomycin (0.050%) in starter, grower and finisher phases (ZBS); T3—feed additive group supplemented with additive MG at concentrations of 0.5% in starter only (MG); and T4—MG and FA supplementation treatment (MGFA) with additive MG at a concentration of 0.5% in starter and additive FA at a concentration of 0.3% in grower and finisher phases (MGFA). All diets were formulated based on wheat, soybean meal, sorghum, and meat and bone meal where the feed additives and phytase were formulated with nutrient and the matrix values, respectively (Table 2 and Table 3). Titanium dioxide was added as an indigestible marker at 0.5% in the grower diets. The diets were made individually based on the formulation of each diet. The nutrient contents of feed ingredients were measured using near-infrared spectroscopy (NIRS, Evonik AminoProx, Germany) before feed formulation. Cold pelleted diets were fed in the starter phase (days 0 to 10; crumbled), grower phase (days 10 to 24) and finisher phase (days 24 to 35) followed by Ross 308 feeding standards for broilers.

### 2.4. Necrotic Enteritis Challenge

The NE challenge model was executed in this study following previously described challenge protocols [23,24] with modification. In brief, on day 9, all birds were orally gavaged with field strains of *Eimeria* spp. oocysts in 1 mL dose consisting of *E*. *acervulina* (5000), *E*. *maxima* (5000), and *E*. *brunetti* (2500) (Eimeria Pty Ltd., Werribee, VIC, Australia). On day 14, all birds were orally gavaged with approximately 10^8^ CFU/mL of *C*. *perfringens* EHE-NE18 strain in 1 mL dose (CSIRO Livestock, Geelong, VIC, Australia).

### 2.5. Performance Measurement

Pen weight and feed intake were recorded on days 0, 10, 24, and 35. Body weights of dead birds were recorded daily and FCR was corrected for the mortalities. Necropsies were carried out to determine the reason for deaths. Dead birds, sampled birds, and birds left at the end of the study (day 35) were opened to further confirm their sex by visual inspection of genital organs.

### 2.6. Sampling and Intestinal Lesion Scoring

On days 16 and 24, two randomly chosen birds (one male and one female) from each pen were weighed, electrically stunned (JF poultry equipment, Weltevreden Park, South Africa), and euthanized by cervical dislocation to collect intestinal samples and perform post mortem analysis. On day 16, cecal contents from two sampled birds per pen were collected in 2 mL Eppendorf tubes and stored at −20 ℃ for microbiota analysis. On day 16, approximately 2 cm of the proximal jejunal tissue from one male bird per pen was excised, flushed with chilled phosphate-buffered saline (PBS), and collected in 2 mL Eppendorf tubes containing RNA later (Invitrogen, Thermo Fisher Scientific, California, USA) and kept at 4 ℃ for 4 h before stored in −20 ℃ for further analysis. On day 24, ileal content from one male bird per pen was collected in 2 mL Eppendorf tube and stored at −20 ℃ for CP and GE digestibility measurements. 

On days 16 and 24, all intestinal sections of sampled birds were excised for NE lesions. Intestinal lesions of the duodenum, jejunum, and ileum were scored by visual examination using a scale ranging from 0 to 6 following a previously described lesion scoring system [25].

### 2.7. Cecal Bacterial Quantification

The cecal bacterial DNA extraction method described by Kheravii et al. [26] was used for this study with minor modifications. The DNA of frozen cecal samples collected on d 16 was extracted using QIAxtractor DNA reagents and QIAxtractor DNA plasticware kits (Qiagen, Inc., Doncaster, VIC, Australia). Approximately 130 mg of defrosted cecal samples and 300 mg of glass beads (0.1 mm) were placed in a 2 mL Eppendorf tube. Then, 300 μL Qiagen Lysis Buffer (270 µL DXL and 30 µL digestive enzyme) was added to Eppendorf tubes containing samples and placed into a bead beater mill (Retsch GmbH and Co., Haan, Germany) at a frequency of 30/S for 5 min. The samples were placed in a heating block and incubated at 55 ℃ for 2 h and followed by centrifugation at 20,000× *g* for 5 min. An aliquot of 200 μL supernatant was placed into the loading block and extraction was carried out using the CAS-1820 Xtractor Gene (Corbett, Sydney, Australia) following the manufacturer’s instruction. In brief, the reactions (DXB, DXW, DXF, or DXE) were placed into assigned locations inside the robotics machine. An aliquot of 400 μL of binding buffer (DXB) was added in the loading block containing 200 μL of supernatant and mixed appropriately, and incubated for 6 min. A volume of 500 μL lysed samples were transferred into the capture columns and vacuumed for 3 min at 30 kPa. An additional 200 μL of DXB was loaded to the capture columns and vacuumed at 35 kPa. Next, an aliquot of 600 μL DXW was added into the capture columns and vacuumed for 2 min at 30 kPa, after that, 600 μL DXF washing buffer was loaded to capture columns and vacuumed for 1 min at 35 kPa and the extracted DNA was dried by vacuuming for 5 min at 25 kPa. At the end of the extraction process, an elution block was applied to elute the cecal DNA by adding 60 μL DXE and elution blocks containing samples were vacuumed for 2 min at 30 kPa. The purity and quantity of the resulting DNA samples were measured with a Nanodrop 8000 spectrophotometer (Nanodrop Technologies, Wilmington, DE, USA). The DNA with ratios of A260/A280 being greater than 1.8 was considered as of high quality and stored at −20 ℃ for further analysis. 

The cecal bacterial DNA quantification methods were applied following previously described procedures [27]. The cecal DNA was diluted 20 times (1:20 dilution) with nuclease-free water and the quantitative real-time polymerase chain reaction (PCR) of six bacterial groups was executed to quantify with a real-time PCR system, Rotorgene 6000 (Corbett, Sydney, Australia). The SYBR-Green containing mix (SensiMix SYBR No-Rox, Bioline, Sydney, Australia) was applied for quantitative polymerase chain reaction (qPCR) and the qPCR was performed in duplicate for each sample. The reaction in an amount of 10 μL contained 2 μL of diluted cecal DNA, 300 mmol/L of forward and reverse primers, and 5 μL of 2 × SensiMix™ SYBR^®^ No-ROX. The genomic DNA copies of *Lactobacillus* spp., *Bifidobacterium* spp., *Bacillus* spp., *Ruminococcus* spp., total anerobic bacteria, and SensiFAST Probe SYBR No-ROX (Bioline, Sydney, Australia) was used for *C*. *perfringens* for the Taqman-based assay. The specific primers used for quantifying these six bacterial groups are presented in Table 4. The target DNA copies were calculated and the quantified bacterial amount was expressed as log_10_ (genomic DNA copy number)/g digesta.

### 2.8. RNA Extraction and cDNA Synthesis

Total RNA from each jejunal tissue sample collected on day 16 was extracted after homogenization in TRIsureTM (Bioline, Sydney, Australia) according to the manufacturer’s instructions. The extracted RNA samples were purified using the Rneasy Mini Kit, (Qiagen, Hilden, Germany) based on the manufacturer’s instructions. The quantity and purity of total RNA samples were measured using a NanoDrop ND-8000 spectrophotometer (Thermo Fisher Scientific, Waltham, USA). An RNA 6000 Nano Kit was applied to determine the RNA integrity number (RIN) using an Agilent 2100 Bioanalyzer (Agilent Technologies, Inc., Waldbronn, Germany). The purified RNA samples were considered as high-quality if the value of 260/230 was higher than 1.8, 260/280 value between 2.0 to 2.2, and the RIN number was greater than 7.0. The isolated RNA of the tissue sample was reverse-transcribed using the QuantiTect Reverse Transcription Kit (Qiagen, Hilden, Germany) according to the manufacturer’s instructions. In brief, one µg of each total RNA sample was incubated at 42 ℃ for 2 min in 2 µL of 7 × genomic DNA (gDNA) Wipeout Buffer to avoid gDNA contamination. After that, the gDNA elimination reaction was added to reverse-transcription reaction components containing one µL of Quantiscript Reverse Transcriptase, 4 µL of 7 × Quantiscript RT Buffer, and one µL of RT Primer Mix and mixed appropriately. The Rotorgene 6000 real-time PCR machine (Corbett, Sydney, Australia) was applied to incubate the mixture at 42 ℃ for 15 min followed by 95 ℃ for 3 min to convert the RNA into cDNA. The cDNA samples were then diluted 10 times with Nuclease-free water and kept at −20 ℃ for further analysis.

### 2.9. Real-Time Quantitative Polymerase Chain Reaction (RT-qPCR)

Amplification and detection were performed in duplicates using an SYBR Green Kit SensiFAST™ SYBR^®^ No-ROX (Bioline, Sydney, Australia) with a Rotorgene 6000 real-time PCR machine (Corbett Research, Sydney, Australia). The PCR reaction was carried out in a volume of 10 µL containing 2 µL of 10 × diluted cDNA template, 400 mM of each primer, and 5 µL of 2 × SensiFAST™ SYBR^®^ No-ROX. A total of eight house-keeping genes, namely, *18S*, *ACTB*, *GAPDH*, *YWHAZ*, *HMBS*, *SDHA*, *HPRT1*, and *TBP*, were used for the optimization of reference genes using the gene expression stability measure (geNorm M) module in qbase+ software version 3.0 (Biogazelle, Zwijnbeke, Belgium). The two most stable house-keeping genes with the lowest M- value (<0.5), *GAPDH* and *HPRT1*, were chosen as optimized reference genes to normalize the expression of the target genes. The amplification cycle (Cq) values for candidate target genes were collected and imported into qBase+ version 3.0 software (Biogazelle, Zwijnbeke, Belgium) and analyzed against the reference genes *GAPDH* and *HPRT1*. The qbase + employed the arithmetic mean method to transform logarithmic Cq values to linear relative quantity, applying the exponential function for relative quantification of genes [33,34] and the output data were exported for the statistical analysis. The normalized relative quantities (NRQ) values were calculated and analyzed across all samples for each target gene. The primers employed in this study were either sourced from previously published studies in chickens or designed using the NCBI Primer-BLAST tool (https://www.ncbi.nlm.nih.gov/tools/primer-blast/ accessed on 6 April 2018) as presented in Table 5. An Agilent 2100 Bioanalyzer (Agilent Technologies, Inc., Waldron, Germany) was used to determine the specificity of each primer pair prior to qPCR analysis using an Agilent DNA 1000 Kit (Agilent Technologies, Inc., Waldron, Germany), and only specific primers amplifying target fragments were used in the qPCR assay.

### 2.10. Apparent Ileal Nutrient Digestibility

Previously stored ileal digesta samples were freeze-dried. The diet and digesta samples were then ground to pass through a 0.5 mm sieve and analyzed for nitrogen (N) content using a combustion analyzer (LECO Corp., St. Joseph, MI). An adiabatic bomb calorimeter (IKA, Werke C7000, GMBH, and Co., Staufen, Germany) with benzoic acid as a calibration standard was applied to determine gross energy (GE) contents of diets and ileal digesta samples in duplicates. A previously described procedure [41] was used to determine titanium dioxide (TiO_2_), an indigestible marker in diets, and digesta samples in duplicates by the colorimetric method. 

Apparent ileal digestibility (AID) of GE and CP (N × 6.25) was determined using the following equation:

$$AID(\%)=(1-\frac{{\mathrm{Diet}\;\mathrm{TiO}}_{2}(\%)}{{\mathrm{Digesta}\;\mathrm{TiO}}_{2}(\%)}\times \frac{\mathrm{Digesta}\;\mathrm{nutrient}\;(\%)}{\mathrm{Diet}\;\mathrm{nutrient}\;(\%)})\times 100$$

### 2.11. Data Analysis

The normally distributed data were subjected to one-way ANOVA analysis using the general linear model procedure of SAS 9.3 package [42] in a completely randomized design. The pen was considered as an experimental unit (n = 32) for the performance data analysis and the values presented in the tables are means with a pooled standard error of the mean (SEM). Performance data were analyzed for the treatment effect with male percentage (corrected to dead birds) set as a covariate. When a treatment effect was detected, the significant differences between means were separated by the Tukey HSD test at the level of *p* < 0.05. Intestinal lesion scores and NE-caused mortality data were analyzed by the non-parametric Kruskal–Wallis test as the data were not normally distributed.

## 3. Results

### 3.1. Bird Performance

The impacts of NE challenge and feed additives on growth performance in broilers are shown in Table 6. One-way ANOVA analysis demonstrated that FCR on days 0 to 10 (*p* < 0.001), 10 to 24 (*p* < 0.001), 24 to 35 (*p =* 0.043), and 0 to 35 (*p =* 0.003), BWG on days 10 to 24 and 0 to 35 (*p* < 0.001 and 0.001, respectively) and FI on days 10 to 24 and 0 to 35 (*p =* 0.011 and 0.036, respectively) showed significant differences. 

In the starter phase (d 0 to 10), birds in the ZBS group had significantly lower FCR compared to all the treatment groups. Body weight gain and FI were not different among the treatment groups. 

In the grower phase (days 10 to 24), birds treated with ZBS had a significantly lower FCR and higher BWG compared to all other treatment groups. Birds fed additives MG and MGFA had a significantly lower FI compared to the ZBS group, but not from the NC group. During the onset of NE (d 10 to 24), BWG and FCR were not significantly different between birds supplemented with feed additives and NC groups. 

In the finisher phase (days 24 to 35), the negative effect of the NE challenge disappeared on BWG and FI from the feed additive and NC groups compared to the ZBS treated group. Birds fed additives (MG and MGFA) had a similar FCR compared to the birds in the NC and ZBS groups, and the NC group had a lower FCR compared to the ZBS group, indicating fast recovery of the surviving birds in the NC and additive groups.

Considering the overall study period (d 0 to 35), birds fed ZBS had a higher BWG and lower FCR compared to all other treatment groups. Birds fed MG had a lower FI compared to the ZBS group, but not different from the NC and MGFA groups. Moreover, the overall bird performance showed that BWG, FI, and FCR were not affected by the diets supplemented with additives MG and MGFA compared to the diet without additive supplementation (NC group).

### 3.2. Necrotic Enteritis Caused Mortality and Lesion Scores

**Figure 1:**
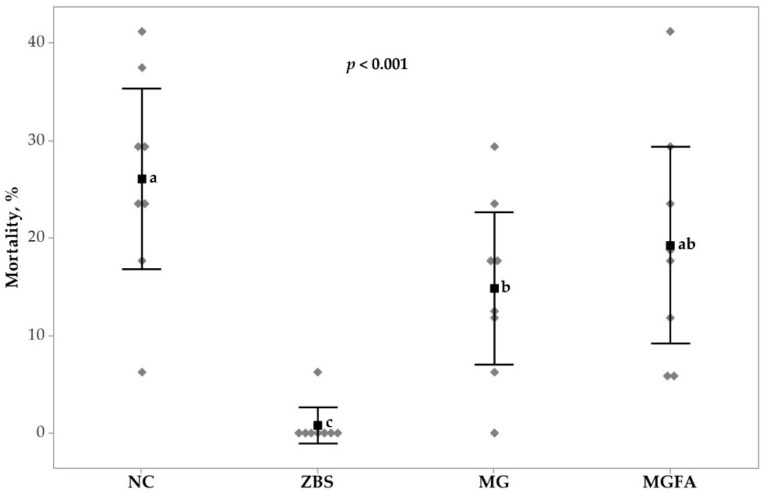
Necrotic enteritis (NE) caused mortality of broilers in response to additive treatments from d 14 to 17. All birds were gavaged with *Eimeria* spp. on day 9 and *C*. *perfringens* on day 14. NC, non-additive control; ZBS, zinc bacitracin and salinomycin; MG, monoglyceride blend; MGFA, MG in starter phase and buffered formic acid (FA) in grower and finisher phases. ^a–c^ values in a row with no common superscripts differ significantly (*p* < 0.05).

**Figure 2:**
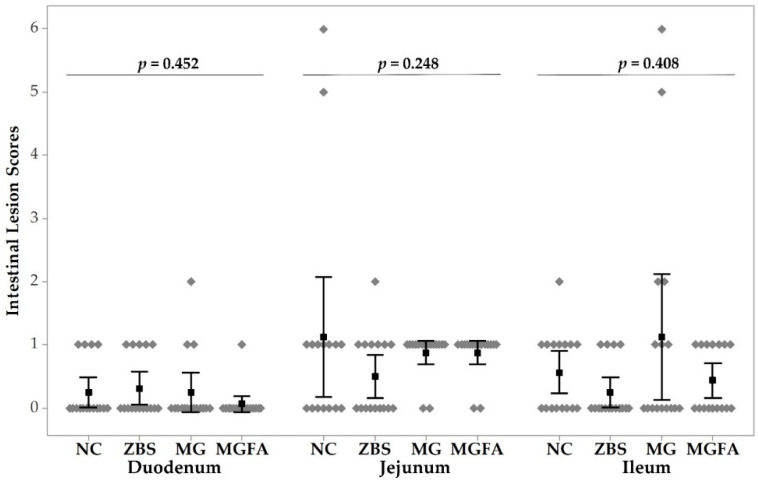
Intestinal lesions of necrotic enteritis (NE) challenged broilers in response to additive treatments on day 16. All birds were gavaged with *Eimeria* spp. on day 9 and *C*. *perfringens* on day 14. NC, non-additive control; ZBS, zinc bacitracin and salinomycin; MG, monoglyceride blend; MGFA, MG in starter phase and buffered formic acid (FA) in grower and finisher phases.

The impacts of NE challenge and feed additives on mortality (days 14 to 17) and lesion scores (day 16) in broilers are shown in Figure 1 and Figure 2. Non- parametric Kruskal–Wallis test showed that mortality due to NE was significantly different (*p* < 0.001). Birds treated with ZBS protected birds from NE caused mortality and had the lowest mortality compared to all other treatment groups, whereas the highest mortality was observed in the NC group. Birds fed additive MG reduced the occurrence of mortality (−11.3%) due to NE compared to the NC group (14.8% vs. 26.1%), but not different from birds in the additive MGFA group. Additionally, birds treated with MGFA showed a numeric reduction of mortality and reduced mortality by 6.8% compared to the NC group (19.3% vs. 26.1%).

Non- parametric Kruskal–Wallis test indicated no significant differences of duodenal, jejunal, and ileal lesions in different treatment groups (*p* = 0.452, 0.248, and 0.408, respectively). Neither birds treated with ZBS nor feed additives (MG and MG+FA) used had any significant effects on intestinal lesions compared to the NC group. Moreover, there were no NE-caused intestinal lesions observed in any of the treatment groups on day 24.

### 3.3. Cecal Bacterial Quantification

The impacts of NE challenge and feed additives on cecal microbiota on day 16 in broilers are presented in Table 7. One-way ANOVA analysis showed that the quantification of *Lactobacillus* spp. and *C*. *perfringens* in cecal content indicated significant differences (*p* = 0.010 and 0.022, respectively). Birds treated with ZBS had lower *Lactobacillus* spp. and not significantly but numerically lower *C*. *perfringens* in the ceca compared to the NC group. Birds fed additive MGFA had a higher amount of *Lactobacillus* spp. compared to the birds in the ZBS group, but not different from NC and MG groups. Birds treated with MG and MGFA had significantly higher *C*. *perfringens* compared to the birds fed ZBS and numerically higher from the NC group. *Bifidobacteria* spp., *Bacillus* spp., *Ruminococcus* spp., and total bacteria were not different between treatment groups.

### 3.4. Expression of Jejunal Genes

The impacts of NE challenge and feed additives on the expression of jejunal genes on day 16 in broilers are presented in Table 8. One-way ANOVA analysis showed the significant differences of *TJP1* (*p* = 0.028), *CLDN5* (*p <* 0.001), *IgG* (*p <* 0.001)*,* and *IgM* (*p* = 0.001) genes in the jejunum, whereas no differences were observed in other genes examined, namely, *OCLDN*, *CLDN1*, *CASP3*, *CASP8*, *E-CADH*, *MUC2*, *MUC5AC*, and *JAM2* (*p* > 0.05). The expression of jejunal *TJP1* was upregulated in the birds fed MG compared to the NC group, but not different from the birds fed ZBS and MGFA. The expression of jejunal *CLDN5* gene was upregulated in the NC and MG groups compared to the ZBS group, but not different from the MGFA group. Birds fed additives MG and MGFA had upregulated *IgG* gene compared to the NC and ZBS groups. The expression of the *IgM* gene was upregulated in the MG and MGFA groups compared to the ZBS group, but not different from the NC group.

### 3.5. Apparent Ileal GE and CP Digestibility

The impacts of NE challenge and feed additives on apparent ileal GE and CP digestibility in broilers on day 24 are shown in Table 9. One-way ANOVA analysis showed that the apparent ileal GE digestibility exhibited significant differences (*p* = 0.013), but no differences of apparent ileal CP digestibility were present among the treatment groups. Birds fed additive MG had a higher GE digestibility compared to the NC group, but not different from the ZBS and MGFA groups.

## 4. Discussion

The clinical form of NE is disastrous to the broiler industry as occurred with impaired performance and high mortality [5,7]. Traditionally, antibiotics have been used to control NE. However, with the ban or phasing out of in-feed AGP from the poultry feed industry, worldwide, there have been concerted efforts to find a comparable alternative to AGP to ameliorate the adverse impacts of NE. Organic acids to some extent have shown to be effective against NE in subclinical form. To be a possible replacement of the in-feed AGP in the commercial broiler industry, it is essential to evaluate the feed additives under more severe diseased conditions. The current study examined the potentials of MG and FA to ameliorate the detrimental impacts of clinical NE on performance, mortality, and intestinal health in broilers. The successful introduction of clinical NE challenge was illustrated by the typical signs of NE observed in birds without supplementation (e.g., presence of intestinal lesions, reduced FI and BWG, increased FCR, and high mortality). Although the challenge was severe in this study, antibiotics were able to protect birds against clinical NE as indicated by very low mortality and improved performance. Results showed that diet supplemented with MG reduced mortality and upregulated *TJP1* and *IgG* genes in the jejunum and improved apparent ileal GE digestibility whereas MGFA fed birds increased the expression of jejunal *IgG* gene compared to the NC group. Altogether, these findings support our hypothesis that birds supplemented with the MG product under investigation improve intestinal health, so provide better protection of the birds from clinical NE indicated by reduced mortality. However, in contrast to our hypothesis, dietary supplementation of FA in grower and finisher phases did not add benefits compared to the MG fed birds alone in the present conditions. Therefore, these results reject our hypothesis that FA supplementation in the grower and finisher phases may add beneficial effects in controlling birds from clinical NE.

The dietary addition of individual OA and their blends in different forms (e.g., calcium, sodium, and potassium salts with or without esterification) have the potential to improve FCR and weight gain, and enhance protection against enteric diseases. In general, the mode of action of OA is believed to be associated with their pH decreasing abilities and antibacterial activities [43]. The OA supplemented in diets mitigate the deleterious effect of enteric diseases on intestinal health via a pH reducing mechanism that decreases pathogenic bacterial load by bactericidal and bacteriostatic activities, resulting in improved microbial inhabitants in the OA supplemented birds compared to the birds without OA supplementation. Supplementation of OA in diets improves intestinal integrity by protecting the disruption of intestinal epithelial cells. Dietary inclusion of OA also improves the digestibility of nutrients by increasing pancreatic enzyme activities [9,44]. Therefore, OA supplemented to diets can have a positive impact on bird performance. On the other hand, monoglycerides are made from the esterification of fatty acids with a glycerol molecule and are harmless without stringent smells by nature. They can be released to the lower part of the intestine via the action of lipase and act against Gram-negative and Gram-positive bacteria [12,45]. Studies have shown that the esterification of fatty acids with glycerol molecules can increase the antibacterial activities, resulting in better microbial inhabitants [14,15]. As a result, monoglycerides supplemented in diets can positively affect bird performance. The current study showed that diet supplemented with MG in the starter phase reduced mortality and increased the expression of *TJP1* and *IgG* genes in jejunum on day 16 and apparent ileal GE digestibility on day 24 compared to the NC group. Birds fed MG in the starter phase and FA in the grower and finisher phases had numerically reduced mortality, significantly increased the expression of jejunal *IgG* gene on day 16 compared to the NC group. These effects indicated the reduced NE severity and improved intestinal health status of birds under clinical NE. However, BWG or FCR were not improved during the entire period of study (days 0 to 35). On the other hand, although FCR was not statistically different between NC and feed additive groups, MG and MGFA treatment groups had lower FCR by 6.0 and 6.8 points compared to the NC group during the onset of NE (days 10 to 24), indicating the positive impact of additive supplementation. Due to the nature of clinical NE present in the current study, the NC group had much higher mortality and thus the entire experimental period showed mostly the performance of survived birds, whose performance was quickly recovered, thus less performance effects of OA were detectable following the recovery. Similar to our results, birds fed blended OA had no effects on BWG but improved FCR under a clinical NE challenge [46] and subclinical NE challenge condition [18]. Moreover, Geier et al. [46] also reported the high mortality in OA-fed birds and not inconsistent from the control group, which is inconsistent with our findings. Reduced mortality in birds fed MG compared to the NC group was possibly due to the higher immune responses and improved gut integrity observed in this study. These improvements of intestinal health in birds fed additives may have a positive impact on nutrient digestibility, indicated by increased apparent ileal GE digestibility. Previous studies have shown that birds supplemented with OA in diets improved apparent ileal GE digestibility [47,48], which further supports our findings. Moreover, additive supplemented birds had similar intestinal lesions compared to birds fed antibiotics, confirming the protective effects of additives as also shown before [47,49]. However, the results observed in this study also revealed that the dietary addition of FA in grower and finisher phases did not have positive impacts on performance and intestinal health over the diet supplemented with MG only in the starter phase as indicated by similar BWG, FCR, intestinal lesions, apparent ileal digestibility, bacterial quantification, and gene expression results in birds fed both additive supplemented diets under the present conditions. Similar to our results, a recent study reported that birds supplemented with FA (high dose) had no effects on performance and mortality in birds under sub-clinical NE challenge [18]. Cumulatively, the findings of this study indicated the beneficial effects of additive MG in reducing the severity of clinical NE on intestinal health, evidenced by improved immunity, gut integrity, digestibility, and reduced mortality. 

An intact intestinal epithelium provides a major defense against the entry of pathogens and maintains homeostasis resulted in proper nutrient digestion, absorption, and utilization, leading to optimal intestinal health and growth performance [50,51]. Tight junction genes such as *CLDN1*, *OCLN*, and *TJP1* are strongly connected with intestinal epithelial cells, and upregulation of these tight junction genes are associated with improved gut barrier integrity and permeability. The expression of *TJP1* gene is correlated with other tight junction genes in the epithelium [52]. Enteric diseases such as NE damage intestinal integrity and downregulates tight junction gene expression (*CLDN*, *OCLN*, and *TJP1*), resulting in increased intestinal permeability [35,53,54]. However, it should be noted that damage in the intestinal epithelium and disturbances in the function of genes regulating tight junctions and immunity can be due to the *Eimeria*-caused infections prior to *C*. *perfringens* challenge of the birds. The application of *Eimeria* spp. prior to the *C*. *perfringens* challenge in the NE challenge model was to predispose birds for the successful induction of NE. Therefore, it is anticipated that the *Eimeria* caused infections would affect the intestinal health of the birds negatively. The results observed in the current study showed that the mRNA expression of tight junction gene *TJP1* was upregulated in birds fed MG compared to the NC group, but not different from ZBS and MGFA fed birds, indicating the potentiality of MG to improve intestinal barrier function, as also shown before in butyrate supplemented birds [55]. Similar to our results, the upregulation of *TJP1* in broilers supplemented with essential oil and OA containing butyric acid was previously reported by Pham et al. [56]. 

Tight junction genes, immunoglobulin genes (e.g., *IgA*, *IgG*, and *IgM*) produced by mucosal plasma cells in the lamina propria are acting as the first line of defense to protect the luminal surfaces and small intestine against diseases [57]. It has been indicated that NE damages intestinal epithelium and lamina propria, resulting in reduced nutrient uptake that in turn impaired immune responses [58]. Wang et al. [59] confirmed the significant effect of NE on immunoglobulin genes evidenced by the reduced expression of *IgA*+ B cells in birds infected with NE. Similarly, Gharib-Naseri et al. [35] reported a reduced expression of *IgG* and *IgM* genes in NE challenged birds. In the current study, upregulation of *IgG* gene in birds fed MG and MGFA compared to the NC group indicates that the birds fed additives had a greater immune response. Furthermore, birds fed MG had upregulated *MUC2* gene (1.306) compared to the NC group (1.093), which is consistent with the reports of Stefanello et al. [60]. *Mucin-2* is the primary mucin produced by goblet cells and is considered a biomarker of intestinal health [61] and higher expression of *MUC2* is known to have improved intestinal health as it protects pathogenic bacterial adhesion to the mucosa [62,63]. Therefore, increased expression of immunoglobulin genes and *MUC2* (despite numerically) in the current study suggests that the additives are able to modulate intestinal health and immune protection under the NE challenged condition. 

Intestinal health plays a key role in nutrient digestion and absorption. The status of the intestinal mucosa is a good indicator of intestinal health. Mucosa plays an important role in the protection of birds against pathogenic bacterial adhesion, resulting in improved barrier function. Previous studies have shown that birds infected with enteric diseases are deemed to have disrupted functions of mucosa and tight junction. Their proper functions are essential for the optimum digestion and absorption of nutrients, thus such disruption results in reduced feed intake and increased energy demands [52,64,65]. Therefore, enhanced tight junction genes and immune responses observed in this study may underlie the improved apparent ileal GE digestibility, as has been shown previously in broilers [48,49]. Moreover, improved apparent GE digestibility in MG fed birds compared to the birds without supplementation could further indicate the ameliorating effects of additive MG against enteric inflammation [66].

## 5. Conclusions

The current study demonstrated that a diet supplemented with monoglyceride blend (MG) has the potential to improve intestinal health and reduce the severity of clinical NE, as indicated by upregulated tight junction and immune genes, improved energy digestibility, and reduced mortality. However, supplementation of FA in grower and finisher phases did not have beneficial effects on the performance and intestinal health over the diet supplemented with MG alone. As expected, the antibiotic was more effective to control the disease outbreak, and thus the development of additives may need to consider a possible combination of different additives with appropriate dosages to improve their usefulness in practice, and recommendations for the poultry industry as alternatives to in-feed AGP. Further research for a better understanding of their mode of action in improved intestinal health and consequent performance, and disease amelioration would provide more information for the poultry industry to combat the challenge posed by antibiotic-free production.

## Figures and Tables

**Table 1:** Treatment groups with additives applied in this study.

Treatments ^1^	Product Name	Inclusion Level; Starter Phase (days 0 to 10), Grower Phase (days 10 to 24) and Finisher Phase (days 24 to 35), %	Necrotic Enteritis Challenge ^2^
NC	-	-	Challenged
ZBS	Zinc bacitracin and Salinomycin	0.033 and 0.05, respectively; in all phases	Challenged
MG	BalanGut^TM^ LS P	Starter: 0.5; Grower and Finisher: 0	Challenged
MGFA	BalanGut^TM^ LS P ** and Amasil^®^ NA *	Starter: 0.5 (MG); Grower and Finisher: 0.3 (FA)	Challenged

^1^ NC, non-additive control; ZBS, zinc bacitracin and salinomycin; MG, monoglyceride blend; MGFA, MG in starter phase and buffered formic acid (FA) in grower and finisher phases. ^2^ All birds were gavaged with *Eimeria* spp. on day 9 and *C. perfringens* on day 14. * Amasil^®^ NA and ** BalanGut™ LS P were provided by BASF, Germany.

**Table 2:** Diet composition used in this study (percentage unless mentioned) ^1^

Item	Starter (days 0 to 10)				Grower (days 10 to 24)				Finisher (days 24 to 35)			
Ingredients ^2^	NC	ZBS	MG	MGFA	NC	ZBS	MG	MGFA	NC	ZBS	MG	MGFA
Wheat	42.0	42.0	42.0	42.0	45.1	45.0	45.1	45.0	50.0	50.0	50.0	50.0
Sorghum	20.0	20.0	20.0	20.0	20.0	20.0	20.0	20.0	20.0	20.0	20.0	20.0
Soybean meal	32.0	32.0	32.0	32.0	28.0	28.0	28.0	28.0	24.0	24.0	24.0	24.0
Meat and bone meal	2.43	2.43	2.43	2.43	1.610	1.608	1.610	1.610	0.862	0.862	0.862	0.862
Cottonseed Oil	0.561	0.550	0.100	0.100	1.900	1.902	1.900	1.901	2.57	2.57	2.57	2.53
Limestone	1.100	1.100	1.100	1.100	1.076	1.075	1.076	1.075	1.065	1.065	1.065	1.065
Salt	0.147	0.147	0.147	0.147	0.196	0.197	0.196	0.167	0.183	0.183	0.183	0.183
Sodium bicarbonate	0.172	0.172	0.172	0.172	0.200	0.200	0.200	0.200	0.240	0.240	0.240	0.166
Sand	0.305	0.230	0.300	0.300	0.300	0.300	0.300	0.300	0.300	0.220	0.300	0.100
Vitamins premix ^3^	0.075	0.075	0.075	0.075	0.075	0.075	0.075	0.075	0.075	0.075	0.075	0.075
Minerals premix ^4^	0.100	0.100	0.100	0.100	0.100	0.100	0.100	0.100	0.100	0.100	0.100	0.100
Choline chloride 60	0.043	0.042	0.040	0.040	0.038	0.039	0.038	0.039	0.033	0.033	0.033	0.033
Kynofos 21P/16Ca	0.100	0.100	0.100	0.100	0.080	0.080	0.080	0.080	0.060	0.060	0.060	0.060
L-lysine HCl	0.385	0.385	0.385	0.385	0.355	0.355	0.355	0.355	0.330	0.330	0.330	0.330
DL-methionine	0.331	0.331	0.331	0.331	0.296	0.296	0.296	0.296	0.262	0.262	0.262	0.262
L-threonine	0.230	0.230	0.230	0.230	0.197	0.197	0.197	0.197	0.171	0.171	0.171	0.171
Phytase (Natuphos^®^ E)	0.020	0.020	0.020	0.020	0.020	0.020	0.020	0.020	0.020	0.0200	0.020	0.020
Monoglyceride blend (MG)	-	-	0.500	0.500	-	-	-	-	-	-	-	-
Buffered formic acid (FA)	-	-	-	-	-	-	-	0.300	-	-	-	0.300
Zinc bacitracin	-	0.033	-	-	-	0.033	-	-	-	0.033	-	-
Salinomycin	-	0.050	-	-	-	0.050	-	-	-	0.050	-	-
Titanium di-oxide	-	-	-	-	0.500	0.500	0.500	0.500	-	-	-	-
Total	100	100	100	100	100	100	100	100	100	100	100	100

^1^ NC, non-additive control; ZBS, zinc bacitracin and salinomycin; MG, monoglyceride blend; MGFA, MG in starter phase following buffered formic acid (FA) in grower and finisher phases. ^2^ Main ingredients were measured using near-infrared spectroscopy (Evonik AminoProx, Germany). ^3^ Vitamin premix/kg diet: vitamin A, 12 MIU; vitamin D, 5 MIU; vitamin E, 75 mg; cyanocobalamin, 0.016 mg; vitamin K, 3 mg; riboflavin, 8 mg; folic acid, 2 mg; nicotinic acid, 55 mg; biotin, 0.25 mg; pantothenic acid, 13 mg; thiamine, 3 mg; pyridoxine, 5 mg; antioxidant Ethoxyquin, 50 mg. ^4^ Mineral premix/kg diet: Cu (sulfate), 16 mg; Mn sulfate and oxide, 60 mg each; I (iodide), 0.125 mg; Fe (sulfate), 40 mg; Se (selenite), 0.3 mg; Zn (oxide and sulfate).

**Table 3:** Nutrient contents of thee diets (as-fed basis, percentage unless mentioned) ^1^.

	Starter (days 0 to 10)				Grower (days 10 to 24)				Finisher (days 24 to 35)			
Nutrients	NC	ZBS	MG	MGFA	NC	ZBS	MG	MGFA	NC	ZBS	MG	MGFA
**Calculated nutrients**												
AME kcal/kg	3000	3000	3000	3000	3100	3100	3100	3100	3200	3200	3200	3200
Crude Protein	24.0	24.0	24.0	24.0	23.0	23.0	23.0	23.0	21.0	21.0	21.0	21.0
Crude fat	2.74	2.73	2.28	2.28	3.96	4.01	3.96	4.00	4.65	4.65	4.65	4.61
Crude fiber	3.15	3.15	3.15	3.15	3.01	3.01	3.01	3.01	2.92	2.92	2.92	2.92
Digestible Arginine	1.370	1.370	1.370	1.370	1.230	1.230	1.230	1.230	1.100	1.100	1.100	1.100
Digestible Lysine	1.280	1.280	1.280	1.280	1.150	1.150	1.150	1.150	1.030	1.030	1.030	1.030
Digestible Methionine	0.604	0.603	0.604	0.604	0.546	0.546	0.546	0.546	0.490	0.490	0.490	0.490
Digestible Methionine+Cystine	0.950	0.950	0.950	0.950	0.870	0.870	0.870	0.870	0.800	0.800	0.800	0.800
Digestible Tryptophan	0.232	0.232	0.232	0.232	0.212	0.212	0.212	0.212	0.200	0.200	0.200	0.200
Digestible Isoleucine	0.912	0.912	0.912	0.912	0.832	0.831	0.832	0.831	0.760	0.760	0.760	0.760
Digestible Threonine	0.860	0.860	0.860	0.860	0.770	0.770	0.770	0.770	0.690	0.690	0.690	0.690
Digestible Valine	1.007	1.007	1.007	1.007	0.923	0.923	0.923	0.923	0.848	0.848	0.848	0.848
Calcium	0.960	0.960	0.960	0.960	0.870	0.870	0.870	0.870	0.790	0.790	0.790	0.790
Phosphorus avail	0.480	0.480	0.480	0.480	0.435	0.435	0.435	0.435	0.395	0.395	0.395	0.395
Sodium	0.160	0.160	0.160	0.160	0.180	0.180	0.180	0.190	0.180	0.180	0.180	0.180
Potassium	0.994	0.994	0.994	0.994	0.913	0.912	0.913	0.912	0.838	0.838	0.838	0.838
Chloride	0.230	0.230	0.230	0.230	0.248	0.24	0.248	0.230	0.230	0.230	0.230	0.230
Choline mg/kg	1700	1700	1700	1700	1600	1600	1600	1600	1500	1500	1500	1500
Linoleic acid	0.888	0.883	0.658	0.658	1.530	1.550	1.530	1.550	1.900	1.900	1.900	1.880
**Analyzed nutrients**												
Gross Energy, kcal/kg	3847	3874	3848	3862	3933	3928	3940	3950	3998	3994	3992	4004
Crude protein	24.7	24.7	24.7	24.6	23.5	23.2	23.3	23.4	21.4	21.2	21.2	21.4
Calcium	0.973	0.962	0.963	0.968	0.879	0.870	0.881	0.881	0.761	0.763	0.769	0.763
Phosphorus	0.687	0.686	0.686	0.698	0.612	0.603	0.610	0.613	0.559	0.542	0.549	0.542

^1^ NC, non-additive control; ZBS, zinc bacitracin and salinomycin; MG, monoglyceride blend; MGFA, MG in starter phase following buffered formic acid (FA) in grower and finisher phases.

**Table 4:** The specific primers applied for quantifying bacteria in cecal contents.

Target Group of Bacteria	Primer Sequence (5′–3′)	Annealing Temperature (℃)	Reference
*Lactobacillus* spp.	F-CAC CGC TAC ACA TGG AG R-AGC AGT AGG GAA TCT TCC A	63	Wise and Siragusa [27]
*Bifidobacterium* spp.	F-GCG TCC GCT GTG GGC R-CTT CTC CGG CAT GGT GTT G	63	Requena et al. [28]
*Bacillus* spp.	F-GCA ACG AGC GCA ACC CTT GA R-TCA TCC CCA CCT TCC TCC GGT	63	Zhang et al. [29]
*Ruminococcus* spp.	F-GGC GGC YTR CTG GGC TTT R-CCA GGT GGA TWA CTT ATT GTG TTA A	63	Ramirez-Farias et al. [30]
*Clostridium perfringens*	F-ATG CAA GTC GAG CGA KG R-TAT GCG GTA TTA ATC TYC CTT T TaqMan Probe-5′-FAM-TCA TCA TTC AAC CAA AGG AGC AAT CC-TAMRA-3′	60	Rinttilä et al. [31]
Total bacteria	F-CGG YCC AGA CTC CTA CGG G R-TTA CCG CGG CTG CTG GCA C	63	Lee et al. [32]

**Table 5:** Sequences of primers used for quantitative real-time PCR.

Item	Sequence	Size (pb)	Annealing T°	Reference
*TJP1*	F-GGATGTTTATTTGGGCGGC R-GTCACCGTGTGTTGTTCCCAT	187	60	Gharib-Naseri et al. [35]
*OCLN*	F-ACGGCAGCACCTACCTCAA R-GGGCGAAGAAGCAGATGAG	123	60	Du et al. [36]
*CLDN1*	F-CTTCATCATTGCAGGTCTGTCAG R-AAATCTGGTGTTAACGGGTGTG	103	60	Gharib-Naseri et al. [35]
*CLDN5*	F-GCAGGTCGCCAGAGATACAG R-CCACGAAGCCTCTCATAGCC	162	61	This study
*JAM2*	F-AGACAGGAACAGGCAGTGCTAG R-ATCCAATCCCATTTGAGGCTAC	135	60	This study
*CASP3*	F-TGGTGGAGGTGGAGGAGC R-GTTTCTCTGTATCTTGAAGCACCA	110	62	Gharib-Naseri et al. [35]
*CASP8*	F-GGAGCTGCTATCGGATCAAT R-GGAGCTGCTCTATCGGATCAAT	126	60	Gharib-Naseri et al. [35]
*IgG*	F-ATCACGTCAAGGGATGCCCG R-ACCAGGCACCTCAGTTTGG	118	60	Zhao et al. [37]
*IgM*	F-GCATCAGCGTCACCGAAAGC R-TCCGCACTCCATCCTCTTGC	98	60	Zhao et al. [37]
*MUC2*	F-CCCTGGAAGTAGAGGTGACTG R-TGACAAGCCATTGAAGGACA	143	60	Fan et al. [38]
*MUC5AC*	F-AAGACGGCATTTATTTCTCCAC R-TCATTACCAACAAGCCAGTGA	244	60	Fan et al. [38]
*HPRT1*	F-ACTGGCTGCTTCTTGTG R-GGTTGGGTTGTGCTGTT	245	62	Yang et al. [39]
*GAPDH*	F-GAAGCTTACTGGAATGGCTTTCC R-CGGCAGGTCAGGTCAACAA	66	61	Kuchipudi et al. [40]

**Table 6:** Performance of necrotic enteritis challenged broilers in response to additive treatments in different phases ^1^.

Treatment ^2^	NC	ZBS	MG	MGFA	SEM	*p*-Value
Starter phase (d 0 to 10)						
BWG, g	270	273	267	266	4	0.423
FI, g	301	298	301	301	4	0.883
FCR	1.117 ^a^	1.090 ^b^	1.128 ^a^	1.134 ^a^	0.006	<0.001
Grower phase (d 10 to 24)						
BWG, g	776 ^b^	938 ^a^	781 ^b^	789 ^b^	15	<0.001
FI, g	1240 ^ab^	1274 ^a^	1192 ^b^	1200 ^b^	18	0.011
FCR	1.590 ^a^	1.362 ^b^	1.530 ^a^	1.522 ^a^	0.024	<0.001
Finisher phase (d 24 to 35)						
BWG, g	1187	1155	1144	1169	20	0.450
FI, g	1805	1824	1756	1787	25	0.272
FCR	1.521 ^b^	1.579 ^a^	1.534 ^ab^	1.531 ^ab^	0.014	0.043
Overall period (d 0 to 35)						
BWG, g	2239 ^b^	2380 ^a^	2202 ^b^	2229 ^b^	28	<0.001
FI, g	3283 ^ab^	3369 ^a^	3220 ^b^	3235 ^ab^	37	0.036
FCR	1.467 ^a^	1.416 ^b^	1.463 ^a^	1.453 ^a^	0.009	0.003

NE = necrotic enteritis; BWG = body weight gain; FI = feed intake; FCR = feed conversion ratio; ^1^ Necrotic enteritis (NE) challenged birds were gavaged with *Eimeria* spp. on day 9 and *C. perfringens* on day 14; ^2^ NC, non-additive control; ZBS, zinc bacitracin and salinomycin; MG, monoglyceride blend; MGFA, MG in starter phase and buffered formic acid (FA) in grower and finisher phases. ^a,b^ values in a row with no common superscripts differ significantly (*p* < 0.05).

**Table 7:** Cecal bacterial loads (log_10_ genomic DNA copies/g digesta) in response to additive treatments in NE challenged broilers on day 16 ^1^.

Treatment ^2^	NC	ZBS	MG	MGFA	SEM	*p*-Value
*Lactobacillus* spp.	8.48 ^a^	7.81 ^b^	8.09 ^ab^	8.61 ^a^	0.17	0.010
*Ruminococcus* spp.	8.79	9.00	8.83	9.03	0.27	0.887
*Bacillus* spp.	6.82	6.77	6.51	7.20	0.28	0.379
*Bifidobacteria* spp.	7.51	7.50	7.68	7.58	0.18	0.893
*Clostridium pefringens*	8.46 ^ab^	7.56 ^b^	8.81 ^a^	8.87 ^a^	0.31	0.022
Total bacteria	10.12	10.09	10.10	10.41	0.15	0.394

^1^ Necrotic enteritis (NE) challenged birds were gavaged with *Eimeria* spp. on day 9 and *C*. *perfringens* on day 14. ^2^ NC, non-additive control; ZBS, zinc bacitracin and salinomycin; MG, monoglyceride blend; MGFA, MG in starter phase and buffered formic acid (FA) in grower and finisher phases. ^a,b^ values in a row with no common superscripts differ significantly (*p* < 0.05).

**Table 8:** The mRNA expression of jejunal genes in response to additive treatments in NE challenged broilers on day 16 ^1^.

Treatment ^2^	NC	ZBS	MG	MGFA	SEM	*p*-Value
*TJP1*	0.902 ^b^	1.018 ^ab^	1.306 ^a^	1.032 ^ab^	0.092	0.028
*OCLN*	1.300	0.895	1.057	1.284	0.209	0.469
*CLDN1*	1.149	0.836	1.301	0.945	0.168	0.230
*CLDN5*	1.059 ^a^	0.632 ^b^	1.097 ^a^	0.840 ^ab^	0.077	<0.001
*JAM2*	1.022	0.789	1.064	0.953	0.093	0.195
*CASP3*	0.923	1.236	1.123	0.780	0.142	0.129
*CASP8*	1.001	1.040	1.180	0.944	0.121	0.569
*E-CADH*	1.132	1.077	1.090	1.014	0.102	0.876
*IgG*	0.937 ^b^	0.997 ^b^	1.496 ^a^	1.456 ^a^	0.106	<0.001
*IgM*	1.150 ^ab^	0.811 ^b^	1.600 ^a^	1.374 ^a^	0.126	<0.001
*MUC2*	1.093	1.163	1.348	1.117	0.168	0.730
*MUC5AC*	1.221	1.122	1.244	1.140	0.127	0.879

^1^ Necrotic enteritis (NE) challenged birds were gavaged with *Eimeria* spp. on day 9 and *C*. *perfringens* on day 14; ^2^ NC, non-additive control; ZBS, zinc bacitracin and salinomycin; MG, monoglyceride blend; MGFA, MG in starter phase and buffered formic acid (FA) in grower and finisher phases. ^a,b^ values in a row with no common superscripts differ significantly (*p* < 0.05).

**Table 9:** Apparent ileal nutrient digestibility of NE challenged broilers in response to additive treatments on day 24 ^1^.

Item	NC ^2^	ZBS	MG	MGFA	SEM	*p*-Value
Gross Energy, %	71.0 ^b^	72.4 ^ab^	75.0 ^a^	73.5 ^ab^	0.008	0.013
Protein, %	81.2	82.3	82.0	80.6	0.010	0.637

^1^ Necrotic enteritis (NE) challenged birds were gavaged with *Eimeria* spp. on day 9 and *C*. *perfringens* on day 14; ^2^ NC, non-additive control; ZBS, zinc bacitracin and salinomycin; MG, monoglyceride blend; MGFA, MG in starter phase and buffered formic acid (FA) in grower and finisher phases. ^a,b^ values in a row with no common superscripts differ significantly (*p* < 0.05).

## Data Availability

The data presented in this study are available on request from the corresponding author.
